# Compensation claims in pediatric orthopedics in Norway between 2012 and 2018: a nationwide study of 487 patients

**DOI:** 10.1080/17453674.2021.1932922

**Published:** 2021-06-04

**Authors:** Joachim Horn, Hanne Rasmussen, Ida Rashida Khan Bukholm, Olav Røise, Terje Terjesen

**Affiliations:** a Division of Orthopaedic Surgery, Section of Children’s Orthopaedics and Reconstructive Surgery, Oslo University Hospital; b Institute of Clinical Medicine, University of Oslo , Oslo; c Department of Orthopaedics, University of Northern Norway; d The Norwegian System of Patient Injury Compensation; e The Norwegian University of Life Science; f University of Oslo and Faculty of Health Sciences, SHARE – Centre for Resilience in Healthcare, University of Stavanger, Stavanger, Norway

## Abstract

Background and purpose — In Norway all compensation claims based on healthcare services are handled by a government agency (NPE, Norsk Pasientskade Erstatning). We provide an epidemiological overview of claims within pediatric orthopedics in Norway, and identify the most common reasons for claims and compensations.

Patients and methods — All compensation claims handled by NPE from 2012 to 2018 within pediatric orthopedics (age 0 to 17 years) were reviewed. Data were analyzed with regard to patient demographics, diagnoses, type of injury, type of treatment, reasons for granted compensation, and total payouts.

Results — 487 compensation claims (259 girls, 228 boys) within orthopedic surgery in patients younger than 18 years at time of treatment were identified. Mean age was 12 years (0–17). 150 out of 487 claims (31%) resulted in compensation, including 79 compensations for inadequate treatment, 58 for inadequate diagnostics, 12 for infections, and 1 based on the exceptional rule. Total payouts were US$8.45 million. The most common primary diagnoses were: upper extremity injuries (26%), lower extremity injuries (24%), congenital malformations and deformities (12%), spine deformities (11%), disorders affecting peripheral joints (9%), chondropathies (6%), and others (12%).

Interpretation — Most claims were submitted and granted for mismanagement of fractures in the upper and lower extremity, and mismanagement of congenital malformations and disorders of peripheral joints. Knowledge of the details of malpractice claims should be implemented in educational programs and assist pediatric orthopedic surgeons to develop guidelines in order to improve patient safety and quality of care.

Patient injuries due to medical care are a large burden for patients and the healthcare system (OECD 2020) leading to increasing attention on patient safety and prevention of medical errors. In a study by de Vries et al. (2008) the median overall incidence of in-hospital adverse events was 9%, many of these being preventable. In Norway all compensation claims based on public and private healthcare services are handled by a government agency, the Norwegian System of Patient Injury Compensation (NPE). One of the tasks of NPE is to contribute with statistical data to improve quality of care and to prevent patient harm. Recent annual reports show that NPE received 5,695 claims in 2020. In the same year 4,917 decisions were made by NPE, and of these 1,481 (30%) resulted in compensation with total payouts of US$135 million. Orthopedic surgery accounted for nearly one-third (n = 1,443) of all claims that were decided in 2020 and 30% (n = 445) of all claims that were granted, resulting in total payouts of US$30 million. Thus, orthopedic surgery is at especially high risk of claims and there is evidence that claims within the field of pediatric orthopedics are more likely to result in payment compensation than adult cases (Orosco et al. 2012, Oetgen and Parikh 2016).

The substantial number of recent publications on medical errors reflect the increasing attention on patient safety, and in 2011 the Norwegian Ministry of Health and Care Services launched “The Norwegian Patient Safety Program: In safe Hands,” a campaign with the aim of reducing patient harm and improving patient safety. This campaign emphasizes that patient injuries are preventable, that increasing attention should be given to patient safety, and that hazards and risks should be identified.

Several papers within different medical subspecialties have been published based on data from the registry provided by the Norwegian System of Patient Compensation (Kongsgaard et al. 2016, Desserud et al. 2017, Fornebo et al. 2017, Norum et al. 2018, Randsborg et al. 2018). So far, no studies have emerged, based on data from NPE, to analyze compensation claims within the field of pediatric orthopedics. In literature indexed in Medline, only 2 studies evaluated patient injuries in pediatric orthopedics (Oetgen and Parikh 2016, Galey et al. 2019). However, these studies provide only an epidemiological overview of the claims. None of the studies evaluated the specific problems that caused the patient injuries, and no concrete recommendations could be given on how to prevent patient harm. Thus, further knowledge of the details of compensation claims is required to help the pediatric orthopedic surgeon to improve patient safety and quality of care.

This study analyzes all malpractice claims within pediatric orthopedics during a certain time period in order to provide epidemiological data, identify the most common reasons for claims and compensations, provide data on specific diagnosis and procedures that led to claims, and to give some recommendations that might help to prevent future of patient injury events.

## Patients and methods

All compensation claims in Norway are handled by NPE. The treating healthcare professional is obliged to inform the patient of the right to seek compensation from NPE. According to information provided by NPE (npe.no), claims are eligible for compensation if 3 conditions are met: (1) the patient injury must be due to treatment failure (treatment error or omission), caused by either examination, diagnosis, or treatment (including follow-up); (2) the patient injury must have resulted in financial loss of more than US$1,165. Despite no financial loss, compensation can be awarded if the patient has sustained a “permanent” and “significant” injury. Permanent would mean the injury lasts for at least 10 years and significant would mean that medical impairment is at least 15% based on a dedicated invalidity table for injuries (Invalidity table 2020); (3) the patient injury must not be too old. The main rule is that the patient must file the claim within 3 years after realizing that an alleged injury has occurred. All claims based on medical care more than 20 years ago are considered to be expired, no matter whether the patient was aware of the injury or not.

However, compensation can also be granted in some exceptional cases even if no error or omission occurred. This rule applies when either infection, which is not caused by the patient’s condition or pre-existing illness, or a particularly severe and unexpected complication occurs.

One of the tasks of NPE is to provide a database of all compensation claims and the basic data on each case. Detailed data on the patient’s treatment and complications and the decision on the matter are prospectively entered into a database in NPE. This database was the basis for this study.

The data was provided in an anonymized version, and no sensitive patient data was included in the database. The database was searched with the terms “orthopedics” and age “0–17 years” for a certain time period (2012–2018). 487 compensation claims within the field of pediatric orthopedics fulfilled the criteria to be evaluated. The number represents all claims within pediatric orthopedics that led to a decision (granted or refused) within the period 2012–2018. The claims were analyzed for the following data: (1) overall number of complaints, patient demographics, age and sex, and number of complaints that resulted in compensation; (2) reason for compensation granted (inadequate examination/diagnostics, inadequate treatment, infection, extensive/major unexpected complication); (3) type of disease/primary injury; (4) conditions with high incidence of compensation claims; (5) geographical region (Northern, Central, Western, South East health region); (6) total payouts. There is no national registry for all pediatric orthopedic conditions in Norway. However, there is a registry for pediatric hip disorders (Barnehofteregisteret 2021) and there is published data available describing the epidemiology of pediatric fractures in a certain area of the country (Randsborg et al. 2013). Data from the pediatric hip registry and extrapolated epidemiological data from the study on pediatric fractures and population data from Statistics Norway (2020) was used to estimate the incidence and risk of claims for certain conditions. Data by Randsborg et al. (2013) is provided for the age group 0–15 years. Therefore, only claims for the same age group were considered for the risk calculation.

### Ethics, funding, and conflicts of interest

Approval by the Regional Ethical Committee (REK) was not required since all data was based on already anonymized records from NPE—data which is provided for quality control studies. The study received no external funding. The authors declare no conflicts of interest.

## Results

**Figure 1.**
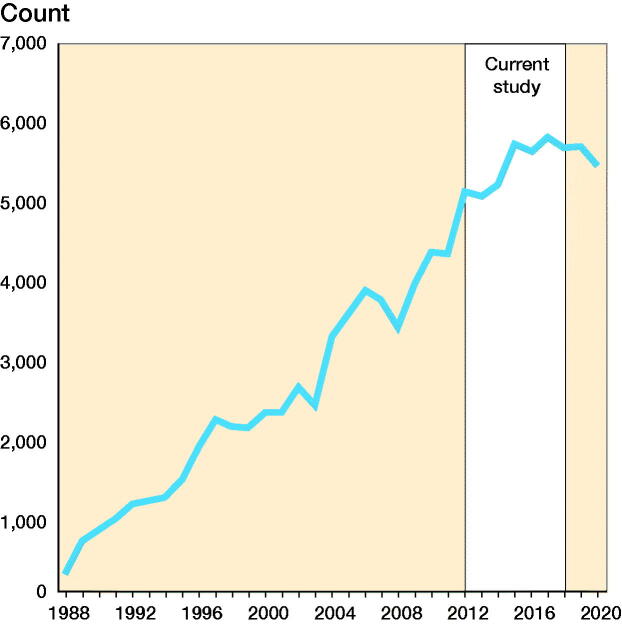
Compensation claims received by NPE from 1988 until 2020. White area represents the time period of the current study (2012–2018).

From 2012 to 2018 (inclusive) NPE received 37,584 compensation claims. Data from NPE shows a continuous increase in compensation claims from 1988, when NPE was established, until 2015, and no further increase in the last 5 years (Figure 1).

Almost one-third (n = 10,861, 29%) of the claims from 2012 to 2018 were within the field of orthopedics, and the second highest number of claims were received for cancer treatment (n = 5183, 14%) (Table 1). 31,440 claims were processed and completed, and 10,264 (33%) of the claims were granted. Within orthopedics 9,596 decisions on claims were made and 3,548 of these were granted (37%).

**Table 1.** Compensation claims received by NPE 2012–2018 according to the 5 most common medical subspecialties

Medical subspecialties	Number (%)
Orthopedics	10,861 (29)
Oncology	5,183 (14)
Dentistry	3,245 (9)
Psychiatry	2,179 (6)
Gastroenterological surgery	1,643 (4)

487 of the claims within orthopedic surgery decided by NPE in this time period apply to children from 0 to 17 years at the time of treatment, including 259 girls and 228 boys. 478 (98%) claims were based on diagnostics and treatment within the public health system, whereas 9 claims applied to the private health system.

The claims refer to possible patient injuries that occurred between 1967 and 2017 and the patients submitted their claims at a mean of 6 years (0–49) after injury. Mean age at time of injury was 12 years (0–17). 150 out of 487 claims (31%) resulted in compensation, including 79 compensations for inadequate treatment, 58 for inadequate diagnostics, 12 for infection, and 1 based on the exceptional rule. No compensation was granted for inadequate follow-up. 16 claims were submitted more than 20 years after the possible patient injury occurred.

Total payouts were US$8.45 million and mean payout for each claim was US$56,331.

The most common primary diagnoses (Table 2) were acute injuries.

**Table 2.** All claims listed in groups according to the ICD 10 classification system and number of claims granted. Values are count

Diagnoses (groups)	Claims	Granted
S40–S69 Acute injuries in the upper extremity and T92 sequela (n = 129)		
S40–S49 Injuries of shoulder and upper arm	51	13
S50–S59 Injuries of the elbow and forearm	49	14
S60–S69 Injuries to the wrist and hand	18	10
T 92 Sequelae	11	2
S70–S99 Injuries in the lower extremity and T93 sequela (n = 115)		
S70–S79 Injuries to the hip and thigh	24	9
S80–S89 Injuries to the knee and lower leg	65	19
S90–S99 Injuries to the ankle and foot	24	6
T93 Sequelae	2	1
Q65–Q79 Congenital malformations and defor-mations of the musculoskeletal system (n = 59) including M16.2–M16.3 secondary arthritis		
Q65 Hip dysplasia and sequelae	36	9
Q66 Congenital deformities of feet	8	1
Q68–Q79	15	2
M40–M54 Deforming dorsopathies, spondylo-pathies, and other dorsopathies (n = 52)		
M40–M43 Scoliosis	44	14
M45–M54	8	1
M20–M25 Arthropathies (n = 46)		
M20 Acquired deformities fingers/toes	8	5
M21–M22 acquired deformities, patella	13	0
M23 Internal derangement of knee	11	7
M24–M25 Other joint disorders	14	5
M91–M94 Chondropathies (n = 30)		
M91 Juvenile osteochondrosis pelvis	5	1
M92 Other juvenile osteochondrosis	5	1
M93 Other osteochondropathies	20	12
Others (n = 56)		
D16, L60, M00, M67, M84, M86, P14, T01, T14 Benign neoplasm, superficial injuries, wounds, pelvic and spine fractures, osteomyelitis	56	18

### Conditions with specifically high rate of compensation claims

Fractures in the distal humerus, femoral fractures, scoliosis, developmental dysplasia of the hip (DHH), and slipped capital femoral epiphysis (SCFE) are among those conditions with the highest number of claims submitted to NPE (Table 3).

**Table 3.** Most common specific diagnoses and information on the number of submitted and granted claims, and reason for granting in children aged 0–17 years. Values are count

		Reasons for granted compensation			
Most common specific diagnoses	Claims	Granted	Diagnostics	Treatment	Exceptional rule
S40–S69 Acute injuries in the upper extremity and T92 sequela (n = 129)					
S42.2 Fractures proximal humerus	5	0			
S42.4 Fractures distal humerus	33	9	1	7	1 **^A^**
S52.5 Distal radius fracture	12	1		1	
S52.4 Antebrachii fractures	11	2		2	
S53.1 Dislocation of elbow	5	2	2 **^C^**		
S62.0 Scaphoid fracture	4	3	2 **^D^**	1	
S62.3-S62.6 Finger/metacarpal fractures	5	4	1 **^D^**	3 **^2 E, 1 F^**	
S63 Subluxation/dislocation, ligament rupture	5	3	2 **^D^**	1 **^F^**	
S70–S99 Injuries in the lower extremity					
S72 Femur fractures	19	7	2	5 **^3 G, 2 H^**	
S82.2 Fracture of shaft of tibia	6	2		2	
S82.3 Fracture lower end of tibia	7	4	2 **^1 D, 1 I^**	2 **^1 J, 1 K^**	
S82.5; S82.6 Fracture medial or lateral malleolus	5	1		1	
S83.5 Sprain of cruciate ligament	13	3	1	1	1 **^A^**
S93 Dislocation and sprain of joints and ligaments at ankle/foot/toe	6	1		1	
M40–M54 Deforming dorsopathies, spondylopathies, and other dorsopathies					
M41.1 Juvenile idiopathic scoliosis	5	1	1		
M41.2 Other idiopathic scoliosis	14	5		3 **^1 L, 2 M^**	2 **^A^**
M41.4 Neuromuscular scoliosis	8	2		2	
Q65–Q79 Congenital malformations and deformations of the musculoskeletal system					
Q65.0–Q65.9 uni- or bilateral DDH	25	8	6	2	
M20–M25 Arthropathies					
M20.1 Hallux valgus	8	5		5 **^4 N,1°^**	
M22.0 Recurrent dislocation patella	5	0			
M23.2 Old meniscus tear	4	2	1 **^D^**		1 **^A^**
M23.5 Chronic instability	6	4		3 **^P^**	1 **^A^**
M91-M94 Chondropathies					
M91.1 Perthes disease	5	1		1	
M93.0 Slipped capital femoral epiphysis (SCFE)	15	10	9 **^D^**	1 **^Q^**	
M93.2 Osteochondritis dissecans	5	2		2 **^R^**	

Detailed information on the causes of compensation for all specific injuries/conditions with > 3 compensations claims submitted to NPE within the time period 2012–2018.

A: infection, B: unexpected severe injury, C: delayed diagnosis of accompanying injuries, D: delayed diagnosis, E: malrotation, F: iatrogenic nerve injury, G: malalignment; H: compartment syndromes in contralateral lower leg due to peroperative hemilithotomy positioning, I: delayed diagnosis compartment syndrome, J: inadequate surgery, K: inadequate conservative treatment; L: iatrogenic spinal cord injury, M: lack of follow-up, N: lack of indication, O: delayed secondary surgery, P: surgical errors, Q: not specified, R: inadequate removal of foreign body, 1 inadequate follow-up.

In relation to the incidence of the conditions, femoral fractures show the highest rate of claims granted within fracture care (0.4%) and all pediatric hip diseases show a relatively high incidence of accepted claims compared with the total occurrence of the specific conditions (DDH = 2%, SCFE = 3.3%, Perthes disease = 0.2%) (Tables 4 and 5).

**Table 4.** Incidence of claims granted in relation to extrapolated occurrence of pediatric fractures in children aged 0–15 years

Type of fracture	Claims granted in children 0–15 years	Incidence per 10^5^ children 0–15 years **^a^**	Extrapolated no.per year **^b^**	for the study period	Incidence of claims granted (%)
S42.4 Fractures of the distal humerus	8	14	1,384	9,688	0.08
S62.3 Finger/metacarpal fractures	2	32	3,110	21,770	0.009
S52.4 Antebrachii fractures	2	9.5	932	6,525	0.03
S72 Femur fractures	3	1	98	686	0.4
S82.2 Fractures of shaft of tibia	2	8.7	853	5,971	0.03

a Epidemiological data published by Randsborg et al. (2013) based on children aged 0–15 years. To allow for extrapolating of data only claims in children of the same age (0–15 years) are considered in this table.

b Incidence of pediatric fractures per 10,000 children (age 0–15 years) was extrapolated to the population of the whole country (981,342 children aged 0–15 years) based on data provided by Statistics Norway (Statistics Norway 2020).

**Table 5.** Incidence of claims for pediatric hip diseases in relation to data provided by the Norwegian National Pediatric Hip Registry

Pediatric hip disease	Claims	Claims granted	Total no.of reported cases^a^	Incidence of claims granted (%)
Q65.0–Q65.9 DDH	23^b^	8	390^b^	2.0
M91.1 Perthes disease	5	1	352	0.2
M93.0 Slipped capital femoral epiphysis	15	10	298	3.3

a Total number of reported cases for the study period are provided by the Norwegian National Pediatric Hip Registry (Barnehofteregisteret 2021).

b The Norwegian National Pediatric Hip Registry provides data for late detected DDH (> 3 months). Only late detected cases are included from both the claim registry and the Norwegian National Hip Registry.

### Geographical distribution of claims (frequency of cases by Health region)

In Norway a state enterprise consisting of 4 regional health authorities is responsible for specialist care, including patient treatment, education of medical staff, and research. The 4 health regions represent geographical regions: South and Eastern, Western, Central, and Northern Norway Regional Health Authority. The distribution of claims according to the health region and based on the population within these regions was, by decreasing order: Northern Health Region (13 per 100,000), Western Health Region (9 per 100,000), Central Health Region (7.7 per 100,000) and South and Eastern Health Region (6.6 per 100,000). Thus, the number of claims per 100,000 inhabitants in Northern Norway was about twice that of the South and Eastern Health Region.

## Discussion

Our study showed that pediatric orthopedics is associated with a high incidence of compensation claims, which confirms findings by other authors, both from the United States (Oetgen and Parikh 2016, Galey et al. 2019). The granting percentage in our material was 31%, comparable to findings by Oetgen and Parikh (2016), who found 33%. Galey et al. (2019) in a US study found a granting percentage of 51%. In the United States there is no centralized, comprehensive malpractice reporting, and numerous databases exist that differ in scope and reporting details (Galey et al. 2019). Although both studies derived from the United States, Galey et al. (2019) found a much higher median indemnity payment than reported by Oetgen and Parkh (2016), a finding which was attributed to the fact that different databases were used in these studies, which might also explain differences in granting percentage.

Claims were granted for inadequate treatment and inadequate diagnosis. In total numbers, upper and lower extremity injuries, arthropathies, chondropathies, and deforming dorsopathies were the most common diagnoses resulting in compensation claims. For those conditions where epidemiological data was available or could be extrapolated, femoral fractures and common pediatric hip disorders (DDH, SCFE, Perthes disease) showed the highest number of claims in relation to the occurrence of these disorders. These findings confirm those by other authors (Oetgen and Parikh 2016, Galey et al. 2019).

Pediatric femoral fractures resulted in a high incidence of claims in relation to their occurrence and in a high rate of granted compensations. The data provided by NPE does not provide sufficient details to draw further conclusions from this finding. However, pediatric femur fractures are rare compared with other pediatric orthopedic fractures (Randsborg et al. 2013), and their treatment might therefore be considered challenging.

It is important to be aware of the risk of compartment syndrome of the contralateral side due to malpositioning (hemilithotomy position) during surgery for femoral fractures. This is an avoidable complication that has been described in the literature (Brouze et al. 2019).

In DDH and SCFE delayed diagnosis was the main reason for granted compensation. Late detected DDH is a frequent problem and there is no consensus on whether clinical examination combined with universal ultrasound or targeted ultrasound improves early diagnostics and treatment outcome (Shorter et al. 2013). Late diagnosis in SCFE is still frequent (Millis 2017). According to the National Norwegian Pediatric Hip registry, SCFE is diagnosed > 6 weeks after onset of symptoms in 70% of patients (Barnehofteregisteret 2021). Healthcare providers should have a high degree of suspicion of SCFE in patients with hip and/or knee and/or thigh pain.

Although the numbers of cases for the different conditions is low, the data might accentuate conditions that deserve special attention. Technical errors were the cause of granted compensation in cases of cruciate ligament surgery and surgery for unstable patella, indicating that this type of surgery might be technically demanding for this age group. In surgery for juvenile hallux valgus, lack of indication was the main cause of compensation. Juvenile hallux valgus is rarely accompanied by symptoms (Hefti 2007) and the management of the condition remains controversial. Surgery might be required only in symptomatic cases. Because the recurrence rate is high (Coughlin 1995), surgery should preferably be postponed until skeletal maturity.

Delayed diagnosis of scaphoid fractures and accompanying injuries to elbow dislocation occurred, indicating that diagnostics in these upper extremity injuries remain challenging (Rasool 2004, Glad et al. 2010).

The fact that several claims were submitted more than 20 years after a possible patient injury reflects that overlooked pediatric orthopedic conditions, such as DDH, might not become apparent and symptomatic until many years later. In these particular cases, the Norwegian jurisdiction to consider compensation claims based on incidents > 20 years ago as outdated might be considered doubtful.

We found a mean payout for each claim of US$∼56,300. Oetgen and Parikh (2016) found an average indemnity payout of US$∼190,000 in pediatric orthopedic patients and Galey et al. (2019) found a median indemnity payment of US$675,000, when only the orthopedic surgeon was named as the defendant. Hence, payouts in the United States were 3 to 12 times higher than in Norway. The huge difference in payouts could be for several reasons. First, in Norway the public sector covers much of the financial needs of an injured patient through public benefits such as social security, sickness benefits, and pensions. The compensation from NPE will cover the difference between income without the injury and income with the injury, when other benefits from the public sector have been taken into account. In the United States, public benefits are much more restricted, and it will vary according to what the individual has in terms of insurance benefits. In addition, compensation in the United States may also be paid as “punitive damages,” compensation that is intended to act as punishment where the tortfeasor has acted negligently. In Norway, such compensation exists only to a very limited extent (personal communication J. Storvik, Senior Advisor, Legal Department, Norsk Pasientkadeerstatning 2020).

Our study showed a higher frequency of compensation claims from the Northern health region than from the other regions. In fact, the number of claims per 100,000 inhabitants from the Northern Health Region was twice the number from the South Eastern Health Region. The NPE data does not offer any reliable explanation for this difference. However, lack of access to specialized pediatric orthopedic care in more remote areas of the country might have an implication for quality of care.

Our study has several limitations. First, the data provided by the registry is limited. Further in-depth analysis of each case would require written consent from the patients. Second, the number of cases within certain diagnoses is relatively low, which limits the strengths of conclusions that can be drawn. Another weakness might be the fact that claims submitted to NPE are evaluated by only one specialist within the field of expertise. The evaluation by the specialist should consider national and international guidelines. However, such guidelines do not exist for many conditions and treatments. Thus, the conclusion concerning the possible existence of patient injuries would to a great extent depend on the personal judgment of only one specialist.

It is a definitive strength of the study that it is population-based because all compensation claims in Norway are handled by NPE. Nevertheless, it must be emphasized that the NPE system does not capture treatment harm when no claim is filed. When NPE was established in 1988, only about 200 patients complained, while in 2018 there were 5,676 complaints. The rise was linear until 2015 and numbers have been more stable in recent years. There is reason to believe that there is still under-reporting. To our knowledge, no previous study on compensation claims within pediatric orthopedics could provide a complete epidemiological overview for a certain time period. Bukholm (2016) found that only 20–35% of claims granted by NPE had been reported to the hospital local registry for adverse events, which means either that hospitals were unaware of approximately 70% of patient injuries or that no reliable system for reporting of patient harm had been established. This underlines the importance of systematically analyzing NPE data for patient safety improvement work.

Our study not only provides epidemiological data, but also analyzed the cases granted in detail to search for recurring patterns of failure as a basis to develop strategies for prevention. This contributes to an increased awareness of patient injuries within pediatric orthopedic care. Knowledge of the details of compensation claims should be part of educational programs for pediatric orthopedic surgeons and assist them to develop and implement guidelines and to improve patient safety and quality of care. On the other hand, efforts to avoid adverse legal outcomes might lead to defensive medicine: a behavior that avoids physician liability without providing increased benefits could lead to possible harm to the patients (Calikoglu and Aras 2020). Guidelines for diagnostics and treatment would assist healthcare workers to keep a balance between anxiety and risk perception.
